# Is the international classification of healthcare procedures (ICHI) a critical point for the implementation of an international Casemix grouper?

**DOI:** 10.1186/1472-6963-12-S1-I1

**Published:** 2012-11-21

**Authors:** Jean-Marie Rodrigues

**Affiliations:** 1 Emeritus PCS International, Saint Etienne Medical School, University of Saint Etienne Jean Monnet, France

## 

Most of developed countries have kept on maintaining, updating and modifying their own coding systems for procedures, as well as national adaptations of ICD, in order to manage and to fund their health care delivery. The most significant efforts were done in Australia with ACHI (Australian Classification of Health Interventions) or ICD10 AM, in Canada with the Canadian Classification of Health Interventions (CCI) developed by the Canadian Institute for Health Information (CIHI) and in France with CCAM (Classification Commune des Actes Médicaux). For some decades several broad pre-coordinated or compositional systems have been proposed to users targeting different goals. The most well known are the UMLS (Unified Medical Language System), LOINC for clinical laboratories, DICOM SDM for imaging, SNOMED, Convergent Medical Terminology (CMT).

Since 2010 WHO FIC has developed ICHI to describe health intervention defined as an activity performed for, with or on behalf of a client(s) whose purpose is to improve individual or population health, to alter or diagnose the course of a health condition or to improve functioning. This definition includes interventions that apply to more than one client or to a population group. As a consequence the prospective international classification will include interventions across the whole health system. It would include interventions provided by all types of providers: doctors, dentists, nurses, allied and community health workers, traditional medicine providers and public health practitioners. The aims of this international classification are to

1) describe and compare the provision and effectiveness of health interventions at the local, national or international level.

2) provide a classification of appropriate scope and detail to which countries may align their more finely grained national or specialty classifications.

3) ensure that a classification is available that can be used without adaptation in countries which do not wish to further refine the classification.

4) take into account that interventions include elements of ‘western’ and ‘traditional’ medicine.

ICHI is now a procedures classification with the level of granularity of ICD-9-CM Volume 3. It is an incentive able to harmonize between the national classifications of health interventions. Such Countries interested in comparability of data including Casemix systems analyzed by an international Casemix grouper should modify their existing systems partially to be compliant with the framework but are not mandated to change the full terminology they use. For countries without an interventions classification and namely developing countries it can be used directly.

ICHI is providing the opportunity to compare the performance of different health care systems using an international Casemix grouper.

